# Stillbirth trends by maternal sociodemographic characteristics among a large internal migrant population in Shenzhen, China, over a 10-year period: a retrospective study

**DOI:** 10.1186/s12889-022-12734-8

**Published:** 2022-02-16

**Authors:** Rui Ma, Lingyun Zou

**Affiliations:** Institute of Women’s and Children’s Health Care, Shenzhen Baoan Women’s and Children’s Hospital, Jinan University, Shenzhen, China

**Keywords:** Stillbirth, Sociodemographic status, Internal migrant population, Maternal birthplace

## Abstract

**Background:**

Cities such as Shenzhen in southern China have large immigrant populations, and the reproductive health issues of pregnant women in these populations have not received sufficient attention. Stillbirth seriously threatens their health and is becoming a social issue worthy of attention. We conducted this study to estimate the trend in stillbirths at 28 or more gestational weeks and the related sociodemographic characteristics of pregnant women among a large internal migrant population in South China.

**Methods:**

A stillbirth is defined as a baby born with no signs of life after a given threshold, and are restricted to births of 28 weeks of gestation or longer, with a birth weight of at least 1000 g for international comparison. A population-based retrospective cohort of all births from January 2010 to December 2019 in Baoan, Shenzhen, was conducted using the Shenzhen Birth Registry Database. The overall stillbirth rate and year-specific stillbirth rate were calculated as the number of foetal deaths ≥28 gestational weeks or a birth weight ≥ 1000 g divided by the number of births over the last decade or in each year, respectively. The associations between the risk of stillbirth and maternal sociodemographic status were assessed using logistic regression. Spearman’s rank correlation was calculated to evaluate the correlation between the economic status of the maternal birthplace and the stillbirth.

**Results:**

An overall stillbirth rate of 4.5 per 1000 births was estimated in a total of 492,184 births in our final analysis. Migrant women accounted for 87% of the total population but had a higher stillbirth rate (4.8 per 1000 births) than the permanent population (2.8 per 1000 births). The stillbirth rate varied by region of maternal birthplace, from 4.1 per 1000 births in women from East China to 5.7 per 1000 births in women from West China. The GDP per capita of the maternal birthplace was strongly correlated with the stillbirth rate.

**Conclusions:**

Large disparities in the stillbirth rate exist between migrant and permanent populations and among regions of maternal birthplace in China. Strategies targeting migrant women based on their maternal birthplace are needed to further reduce the burden of stillbirth.

**Supplementary Information:**

The online version contains supplementary material available at 10.1186/s12889-022-12734-8.

## Background

The stillbirth rate (≥28 weeks’ gestation) is estimated to be 18.4 per 1000 births globally [1]. However, the burden of stillbirth is unequal, with 84% of all stillbirths in the world occurring in lower-middle and low-income countries [2]. China approximately ranks as the fourth highest number of stillbirths in the world with large regional variations in the burden of stillbirths [3].

Since the economic reform in the 1980s, China has experienced decades of rapid urbanization with large- scale rural-to-urban and west-to-east intercity migration [4]. Although driving fast economic growth, the internal migration has given rise to an expansive inequality in sociodemographic development and health care provision between eastern and western provinces and between urban and rural areas in China [5]. Internal migrants account for nearly 20% of the total population in China but cannot hold hukou registration in the city they work due to China’s permanent residence registry system (called *hukou* in Chinese) [ 6]. They usually have to pay out-of-pocket expenses for medical services in cities due to lack of health reimbursement under the hukou-based health insurance system, which exposes them and their children to higher health risks [7, 8].

As the first special economic zone located in the Pearl River Delta of South China, Shenzhen has undergone rapid urbanization with millions of labour migrants since the beginning of the 1980s [9]. Baoan epitomizes this urbanization with approximately 82% internal migrants from various regions of China [10]. Studies have shown that migrating populations in Shenzhen are faced with limited health care options and are at a higher risk for maternal and infant health issues [11–13]. However, little is known about the sociodemographic status and stillbirth trends of the internal migration population in China.

The present study aims to fill this gap based by analysing the data of all births in Baoan during 2010–2019 registered in the Shenzhen Birth Registry Database. We assessed temporal trends in the stillbirth rate and the associated maternal sociodemographic factors. Disparities in maternal sociodemographic status and stillbirth rates were evaluated between migration and urban populations, as well as between western and eastern regions on the basis of maternal birthplace. Finally, we estimated the correlation between the economic status of the maternal birthplace and the risk of stillbirth to fully depict the impact of sociodemographic factors on the burden of stillbirth.

## Methods

### Study design and population

This cohort study was based on data of all births in Baoan from 1 January 2010 to 31 December 2019, extracted from the Shenzhen Birth Registry Database, which has served as a system for birth registration and maternal and infant health management since 2000 [14]. Maternal sociodemographic characteristics and infant clinical records were available for the identification of stillbirth and associated factors.

### Ethics and consent

The study was performed in accordance with the Declaration of Helsinki and was approved by the Ethics Committee of Shenzhen Baoan Women’s and Children’s Hospital under the protocol number LLSC-2020-03-05.The requirement to obtain any informed consent was waived by the Ethics Committee of Shenzhen Baoan Women’s and Children’s Hospital, because only routine maternity data in the China Birth Registry System were used and the patients/the public were not involved in the design, conduct, reporting, or dissemination plans of our research. Data collected were anonymous, and no individually identifiable information were available in the analysis.

### Definitions

We restricted our analysis to births of 28 completed weeks of gestation or longer, or birth weights of at least 1000 g to conform to the WHO definition of third trimester stillbirths [1, 2, 15]. Maternal sociodemographic characteristics (age at delivery, education, migration status, birthplace, trimester of first prenatal care visit and number of prenatal care visits) were selected based on the scientific literature [16, 17]. Maternal age at delivery was categorized into four groups: ≤15, 16–20, 21–35, and ≥ 36 years and maternal educational attainment was classified as primary school and below, middle school, high school, and college and above [16]. Three types of migration status were recorded in the system: permanent (women with hukou registered in Shenzhen), temporary (women living in Shenzhen for 1 year or longer but without hukou registration) and floating (women living in Shenzhen for less than 1 year and without hukou registration). The permanent population is entitled to health care insurance under the permanent residence registry system (*hukou*), while the temporary population receives selected welfare and floating migrants lack most of the social benefits from the local government [18]. Three trimester groups were generated based on the gestational age at the first prenatal care visit [19]. The number of prenatal care visits was transformed into the prenatal care utilization rate, by calculating the ratio between the actual number of visits and the recommended number of visits [19]. The ratio was then classified into four groups: inadequate (< 50%), intermediate (50–< 80%), appropriate (80–< 110%) and adequate plus (≥110%) [20]. To evaluate the disparities in sociodemographic characteristics and stillbirth rates by maternal birthplace at the provincial level, we calculated the number of migrants, stillbirth rate, average gross domestic product (GDP) per capita, average maternal age, proportion of college and above maternal education attainment, and proportion of intermediate and above prenatal care utilization for each province of maternal birthplace. The average GDP per capita for each province of maternal birthplaces in China during 2010–2019 was calculated to measure provincial economic status based on GDP data collected from the National Bureau of Statistics of China [21]. We further classified these provinces into 5 GDP quantile groups based on their average GDP per capita in recent decades: <Q1($5609), Q1($5609)–<Q2($6093), Q2($6093)–< Q3($7297), Q3($7297)–<Q4 ($10,356), and ≥ Q4 ($10,356). To evaluate the disparities in sociodemographic characteristics and stillbirth rates by maternal birthplace at the regional level, provinces from mainland China were divided into three regions (west, central, and east) according to China’s standard regional definition based on disparities in economic progress since 1986 [22].

### Statistical analysis

The overall stillbirth rate and year-specific stillbirth rate were calculated as the number of fetal deaths ≥28 gestational weeks or birth weight ≥ 1000 g divided by the number of births over the last decade or in each year, respectively [1]. Multivariable binomial logistic regression was applied to assess adjusted odds ratios (AORs) and 95% confidence intervals (95% CIs) of both time trends in stillbirth rate and maternal sociodemographic characteristics for stillbirth [23, 24]. Spearman’s rank correlation coefficients were calculated to measure the relationships between stillbirth rate and maternal sociodemographic characteristics with 31 maternal birthplace provinces in mainland China [25]. Average maternal age, percentage of college and above maternal education attainment, and the proportion of intermediate and above prenatal care utilization were calculated for each maternal birthplace in the Spearman’s rank correlation analysis. The stillbirth rate was excluded in Spearman’s rank correlation analysis when the material birthplace contained less than 300 migrants or had a stillbirth rate under 1. All analyses were conducted using Python software (V.3.6.6; Python Software Foundation). Alpha levels of 0.001, 0.01 and 0.05 indicated statistical significance for a two-tailed test separately [26]. Missing values of several variables were included in the descriptive analysis but removed from the logistic regression analysis.

## Results

### Stillbirth rates and maternal sociodemographic status

During 2010–2019, 492,659 infants were born in Baoan, Shenzhen. We excluded 187 births (0.04%) with unclear foetal outcomes, 288 births (0.06%) with a gestational age less than 28 weeks or an unknown age, and birth weights less than 1000 g or an unknown weight, leaving 492,184 births (99.9%) for the final analysis (Supplementary Fig. S1). There were 2228 stillbirths, giving an overall stillbirth rate of 4.5 per 1000 births. The distribution of total births and stillbirths by maternal sociodemographic characteristics is shown in Table 1. Eighty-seven percent of the total population was migrant, with 60% belonging to the temporary population and 27% to the floating population. The stillbirth rate among the floating population (5.7 per 1000 births) was significantly higher than that among the temporary population (4.4 per 1000 births) and the permanent population (2.8 per 1000 births). The risk of stillbirth with young maternal age (≤ 15 years) was over 4 times higher than for the maternal age between 21 and 35 (adjusted OR (AOR): 4.1, 95% CI: 2.0–8.4, *P* < 0.001). Births with maternal education of primary school and below had a significantly higher stillbirth rate than those with a college education and above (adjusted OR (AOR): 2.0, 95% CI: 1.5–2.7, *P* < 0.001). The stillbirth rate in the floating population was nearly double that in the permanent population (adjusted OR (AOR): 1.5, 95% CI: 1.3–1.9, *P* < 0.001). Women from the lowest GDP per capita group had a significantly higher risk of stillbirth than those from the highest GDP per capita group (adjusted OR (AOR): 1.6, 95% CI: 1.1, 2.3, *P* < 0.01). Births with first prenatal care visits in the third trimester had a higher risk of stillbirth than those in the first trimester group (adjusted OR (AOR): 1.2, 95% CI: 1.2–1.4, *P* < 0.001). A total of 84.4% of women had not reached the recommended number of prenatal care visits, and over one-third of women had inadequate prenatal care utilization. The intermediate prenatal care utilization group (50–< 80%) had the lowest stillbirth rate (2.8 per 1000 births), while the inadequate group had the highest stillbirth rate (5.9 per 1000 births) (adjusted OR (AOR): 2.7, 95% CI: 2.3–3.0, *P* < 0.001).

**Table 1:** Associations between maternal sociodemographic characteristics and stillbirth rates in Baoan, Shenzhen, 2010–2019

	Number of births (%)^a^	Stillbirths per 1000 births (‰)^b^	AOR (95% CI)^c^	***P*** value
**Total**	492,184 (100.0)	2228 (4.5)	–	–
**Year of delivery**				
**2010**	42,057 (8.5)	190 (4.5)	reference	–
**2011**	46,923 (9.5)	226 (4.8)	1.1 (0.9–1.4)	0.226
**2012**	55,288 (11.2)	240 (4.4)	1.2 (1.0–1.4)	0.08
**2013**	47,118 (9.6)	190 (4.0)	1.5 (1.2–1.9)	< 0.001
**2014**	50,334 (10.2)	236 (4.7)	1.8 (1.5–2.2)	< 0.001
**2015**	46,093 (9.4)	215 (4.7)	1.8 (1.5–2.2)	< 0.001
**2016**	51,542 (10.5)	214 (4.2)	1.7 (1.4–2.1)	< 0.001
**2017**	52,997 (10.8)	207 (3.9)	1.6 (1.3–1.9)	< 0.001
**2018**	49,235 (10.0)	252 (5.1)	2.1 (1.7–2.6)	< 0.001
**2019**	50,597 (10.3)	258 (5.1)	1.8 (1.4–2.2)	< 0.001
**Maternal age (year)**				
≤ 15	362 (0.1)	8 (22.1)	4.1 (2.0–8.4)	< 0.001
16–20	21,753 (4.4)	190 (8.7)	1.7 (1.5–2.0)	< 0.001
21–35	429,538 (87.3)	1790 (4.2)	reference	–
≥ 36	40,531 (8.2)	240 (5.9)	1.4 (1.2–1.6)	< 0.001
**Maternal education**				
Primary school and below	8699 (1.7)	62 (7.1)	2.0 (1.5–2.7)	< 0.001
Middle school	141,100 (28.7)	749 (5.3)	1.7 (1.5–2.0)	< 0.001
High school	211,654 (43.0)	1042 (4.9)	1.6 (1.4–1.8)	< 0.001
College and above	130,731 (26.6)	375 (2.9)	reference	–
**Migration status**				
Permanent population	64,439 (13.1)	180 (2.8)	reference	–
Temporary population	293,842 (59.7)	1286 (4.4)	1.4 (1.2–1.7)	< 0.001
Floating population	133,903 (27.2)	763 (5.7)	1.5 (1.3–1.9)	< 0.001
**GDP group of maternal birthplace**^d^				
< Q1	60,961 (12.4)	375 (6.2)	1.6 (1.1–2.3)	< 0.01
Q1 – < Q2	63,881 (13.0)	302 (4.7)	1.4 (1.0–2.0)	0.064
Q3 – < Q4	252,881 (51.4)	1038 (4.1)	1.3 (0.9–1.8)	0.124
≥ Q4	10,687 (2.2)	35 (3.3)	reference	–
Missing	1019 (0.2)	8 (7.9)	–	–
**Time of first visit**				
First trimester	375,699 (76.3)	1464 (3.9)	reference	–
Second trimester	62,472 (12.7)	377 (6.0)	1.2 (1.1–1.4)	< 0.001
Third trimester	54,013 (11.0)	387 (7.2)	1.3 (1.2–1.5)	< 0.01
**Prenatal care utilization rate**^e^				
< 50%	185,832 (37.8)	1090 (5.9)	2.7 (2.3–3.0)	< 0.001
50% – < 80%	175,929 (35.7)	498 (2.8)	reference	–
80% – < 110%	53,552 (10.9)	210 (3.9)	1.6 (1.3–1.9)	< 0.001
≥ 110%	76,871 (15.6)	430 (5.6)	2.6 (2.3–2.9)	< 0.001

^a^Distributions of maternal characteristics among the whole study population were calculated by the number of births with women in each subcategory divided by the total number of births, 492,184

^b^Overall and subtype stillbirth rates were calculated by the number of third trimester stillbirths (foetal deaths ≥28 weeks or birth weight ≥ 1000 g) divided by the number of births in each maternal subcategory

^c^AOR, adjusted odds ratio of risk factors in the multivariable binomial logistic regression model, after removing 1019 records due to missing values in any risk factor

^d^The GDP per capita group of maternal birthplaces is generated by classifying maternal birthplaces by the average GDP per capita during 2010 and 2019

^e^The prenatal care utilization rate is defined as the ratio between the actual number of visits and the recommended number

### Secular trends in the stillbirth rate

**Fig. 1:**
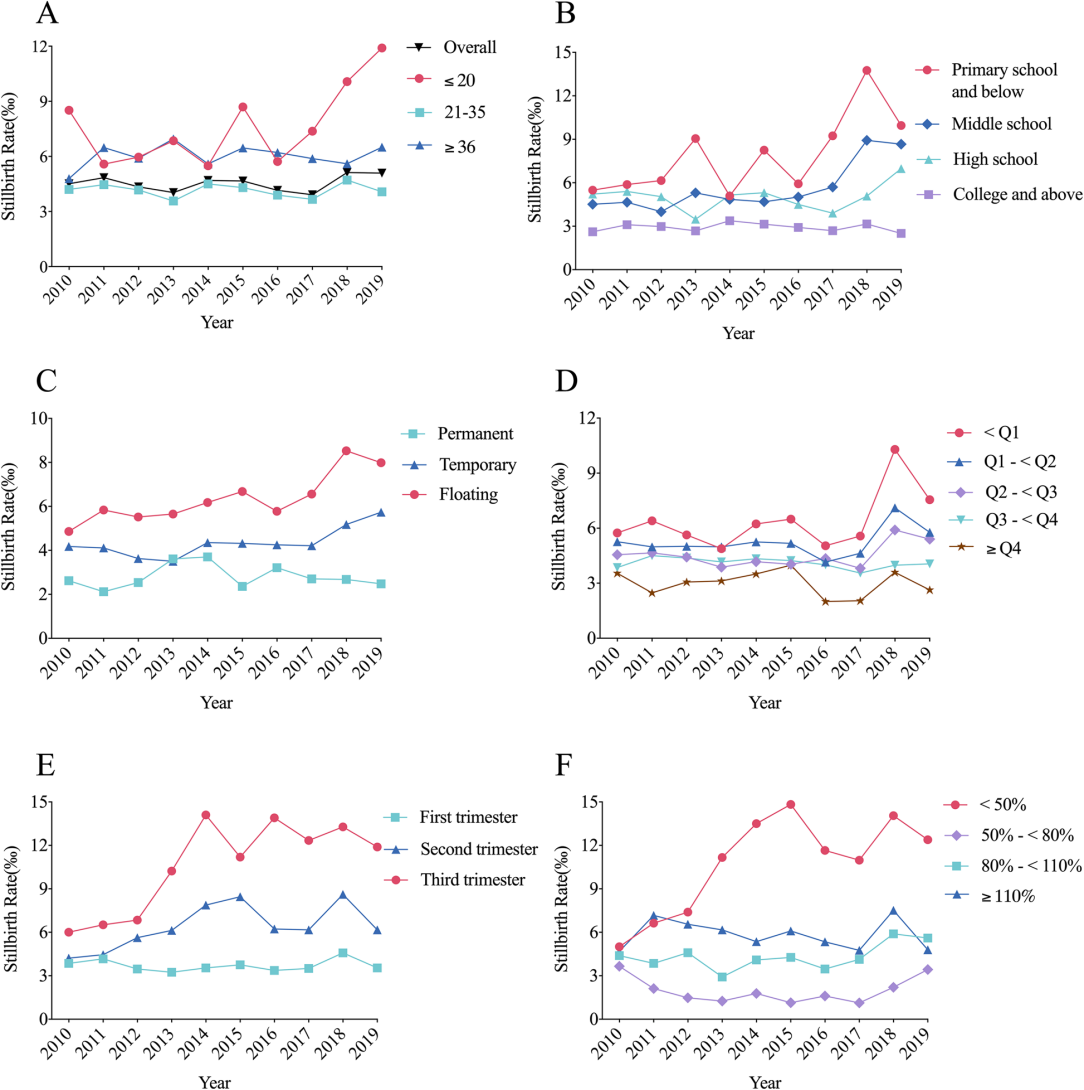
Secular trends in the stillbirth rate among 492,184 births (475 ineligible birth records were excluded) sub-\categorized by maternal sociodemographic characteristics in Baoan, Shenzhen, 2010–2019. **A** Maternal age, **B** maternal education, **C** maternal migration status, **D** GDP per capita group of maternal birthplaces, **E** trimester of first prenatal care visit and **F** prenatal care utilization group

The annual stillbirth rates in Baoan Shenzhen during 2010–2019 fluctuated between 3.9 and 5.1 stillbirths per 1000 births. No monotonous linear trend in the annual stillbirth rate was observed in Table 1. The secular trends of stillbirth rate for each maternal sociodemographic subgroup in this study are shown in Fig. 1 (Supplementary Table S1). Births with a maternal age under 21 or over 35 (Fig. 1A), lower education attainment (Fig. 1B), a floating or temporary migration status (Fig. 1C), lower GDP per capita group of maternal birthplace (Fig. 1D), a later first prenatal care visit (Fig. 1E) and lower prenatal care utilization rate (Fig. 1F) had higher risks of stillbirth over the last decade in Baoan, Shenzhen.

### Disparities in maternal sociodemographics by migration status

Disparities in maternal sociodemographic characteristics among the three maternal migration statuses are presented in Fig. S2 (Supplementary Table S2). The migrant population had a lower level of maternal sociodemographic status than the permanent population in Baoan, Shenzhen. In addition, the floating population had the lowest sociodemographic status among three groups and had the highest percentage of women below 21 years old (7.1%, Fig. S2A), with an education attainment below high school (37.3%, Fig. S2B), a birthplace with GDP per capita less than Q1 ($5609) (15.7%, Fig. S2C), a birthplace located in the west of China (25.0%, Fig. S2D), a first prenatal care visit in the third trimester (24.0%, Fig. S2E) and inadequate prenatal care utilization (42.0%, Fig. S2F).

### Disparities in maternal sociodemographics and stillbirth rates by maternal birthplace

**Fig. 2:**
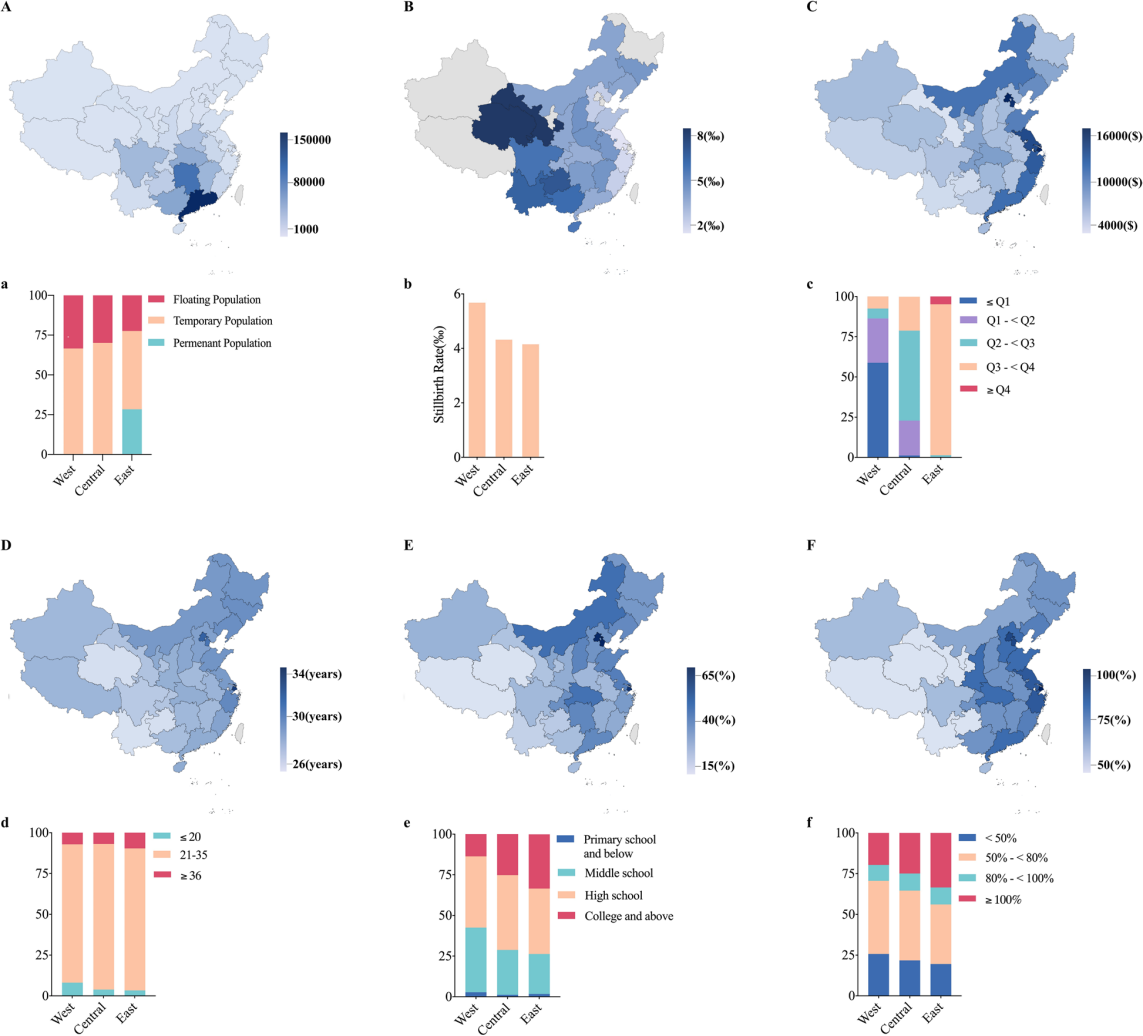
Disparities in stillbirth rates and maternal sociodemographic characteristics by maternal birthplace at the provincial (A-F) and regional (a-f) levels. **A** Number of migrant women (without permanent residency), **B** stillbirth rate, **C** average GDP per capita, **D** average maternal average age, **E** proportion of college and above maternal education, **F** proportion of intermediate and above prenatal care utilization; (a) maternal migration status, (b) stillbirth rate, (c) 5-quantile groups of average GDP per capita, (d) maternal age group, (e) maternal education group, (f) prenatal care utilization group. * Provinces from mainland China were divided into three regions (west, central, and east) according to China’s standard region definition based on disparities in economic progress since 1986

Disparities in maternal sociodemographic characteristics and stillbirth rates by maternal birthplace in Baoan from 2010 to 2019 are presented in Fig. 2 (Tables S3, S4). The migrant population (temporary or floating population) mainly came from provinces near Shenzhen, especially Guangdong Province which accounted for 33.4% of the total migrants (Fig. 2A). The proportion of the migrant population varied from 71.6% in eastern China to 99.7% in central or western China (Fig. 2a**)**. The stillbirth rates ranged from the lowest at 1.3 stillbirths per 1000 births among women from Jiangsu Province to the highest at 8.8 stillbirths per 1000 births among women from Gansu in China (Fig. 2B, Table S3); and from 4.1 stillbirths per 1000 births among women from eastern China to 5.7 stillbirths per 1000 births among women from western China (Fig. 2b, Table S4). Provinces near the east coast of China generally had higher GDP per capita than provinces in the central and western China (Fig. 2C, c, Table S3, S4). The average maternal age at delivery varied between provinces and regions, from 26.1 in Yunnan (located in western China) to 33.3 in Shanghai (located in eastern China) (Fig. 2D, Table S3), and the eastern region had a larger average maternal age at delivery than the central and western regions (Fig. 2d, Table S4). The proportion of women with a maternal education attainment level of college and above was highest at 71.2% and lowest at 10.4% among different provinces in mainland China (Fig. 2E, Table S3) and the western region had the highest proportion of women with an education attainment lower than college (Fig. 2e, Table S4). The proportion of intermediate and above prenatal care utilization ranged from 45.6 to 95.6% (Fig. 2F, Table S3), and the highest proportion of inadequate prenatal care utilization occurred in the western region (Fig. 2f, Table S4).

### Correlation between maternal birthplace and stillbirth rate

In Table 1, we examined the association between GDP per capita of the maternal birthplace and the risk of stillbirth. To further analyze the impact of maternal birthplace on stillbirth, we determined the provincial GDP per capita, maternal average age, education attainment and prenatal care utilization by each maternal birthplace. The Spearman’s coefficients in Supplementary Table S5 indicated that women from provinces with a higher economic status had a higher average age at delivery (Spearman’s coefficient: 0.754; *P* < 0.001), a higher education level (Spearman’s coefficient: 0.792; *P* < 0.001), more sufficient prenatal care (Spearman’s coefficient: 0.751; *P* < 0.001) and finally, a lower risk of stillbirth (Table 1).

## Discussion

### Main findings

A total of nearly half of a million births were delivered, 87% of which were by migrant women in Baoan Shenzhen over the last decade. The stillbirth rate was 4.5 stillbirths per 1000 births, fluctuating between 3.9 and 5.1 per 1000 births from 2010 to 2019. The stillbirth rate varied by maternal migration status, from 2.8 per 1000 births in the permanent population to 5.7 per 1000 births in the floating population. It also varied by region of maternal birthplace, from 4.1 per 1000 births in women from East China to 5.7 per 1000 births in women from West China. Specifically, the stillbirth rate in Baoan Shenzhen during 2010 and 2019 was found to be inversely correlated with GDP per capita of maternal birthplace, with a Spearman’s coefficient of − 0.875.

### Strengths and limitations

There are several limitations in our study. First, stillbirth was defined as third trimester fetal death (gestational age ≥ 28 weeks or birth weight ≥ 1000 g). Stillbirths less than 28 gestational weeks were excluded in this study, therefore we are unable to provide a wider spectrum of stillbirth risk in different gestational periods [2]. Furthermore, detailed information used to measure maternal sociodemographic status, such as occupation, household income and health care insurance, were not collected in the database. A comprehensive and in-depth assessment of the effect of maternal sociodemographic factors on stillbirth cannot be analyzed. Finally, important risk factors for stillbirth other than maternal sociodemographic status, such as obstetric complications, were not considered in our study [16].

Our study also has numerous strengths. To the best of our knowledge, this is the first study in China to focus on a large number of internal migrants and investigate associations between maternal sociodemographic characteristics and the risk of stillbirth among them over a long period of time. Furthermore, a quantitative relationship between the economic status of maternal birthplaces and the stillbirth rate measured by provincial GDP per capita has never been explored before. We also addressed the regional disparity in economic development and its effect on maternal sociodemographic status, and finally, the impact on the stillbirth rate in China based on maternal birthplace.

### Interpretation

With the same definition of stillbirth (fetal deaths of 28 completed weeks of gestation or a birth weight of at least 1000 g), the stillbirth rate in Baoan Shenzhen was slightly lower than the national rate of 5.5 and the global rate of 13.9 stillbirths per 1000 births in 2019 [2]. However, it was still higher than those of high-income counties, including Denmark, Finland, Switzerland, and Sweden [2]. The stillbirth rate in Baoan Shenzhen has remained relatively stable, and remarkable progress in reducing the stillbirth rate in China was reported during 2010 and 2019 [2].

China has experienced an increasing and massive internal migration from rural to urban areas with a rapid urbanization process over the last 40 years since the economic reform [27, 28]. However, the massive migration has posed challenges for maternal and infant health care providers in host cities [29, 30]. There were large disparities in maternal sociodemographics and the risk of stillbirth between the migrant population and the permanent population in Baoan Shenzhen. These disparities can be further subdivided by maternal birthplaces at both the provincial and the regional levels, with the economic development as a driving factor. There were significant gaps in economic development in different provinces and regions in China. Our study highlights that paralleling the decrease in the average GDP per capita of maternal birthplaces, migrant women from provinces with lower levels of economic development (usually located in western China) had lower sociodemographic characteristics, including a lower age at delivery, a lower education level and lower prenatal care utilization. These sociodemographic disparities have resulted in the persisting unequal burden of stillbirths between migrant and permanent populations, and among different birthplace provinces and regions over the last decade.

Similar economic and sociodemographic disparities have been demonstrated by many studies among the international migrant population, especially in industrialized countries [31–34]. A 10-year ecological study of 20 countries in Latin America found a similar relationship between GDP per capita of maternal country of origin and stillbirth rate with a Spearman’s coefficient of − 0.8226 (*p* < 0.001) [33]. Uncoordinated economic development has been observed between western and eastern areas in China for many years [22, 35].

Regional gaps in economic development have not only affected women’s education attainment, and age at delivery but also access to health care resources and prenatal care utilization particularly in the rural and remote regions of western China [36–38]. Children growing up in lower-income families are more likely to drop out of school and tend to lag behind in their education [39]. Family income and school attainment have been found to be important determinants of a woman’s age at marriage and at first birth [40]. Young age at delivery (less than 20 years old) has been reported as a significant risk factor for stillbirth [1]. Meanwhile, significant inequality in the geographic distribution of health care resources exists in China in line with economic inequality, and women from higher economic backgrounds tend to have higher utilization of health resources than other groups [41, 42].

Adequate prenatal care is important for maternal and infant health and health insurance plays an essential role in the utilization of prenatal care services [43, 44]. However, prenatal care utilization among migrants has been reported to be significantly lower than that among non-migrants in both our study and other studies [11, 31]. Effective, region-unrestricted health insurance coverage and free prenatal care services for migrant women, especially those from rural western regions need to be considered by local and central governments to further reduce the stillbirth burden across the country [44]. In addition, by improving the working environment and treatment of women, such as implementing equal pay for equal work regardless of residence status, the stillbirth risk of migrant women may also be reduced [45]. However, the recommendation for prenatal care visits in Shenzhen is much more intense than the recommendations by WHO. The number of prenatal care recommended by WHO is about 8 (including 4 ultrasound examinations), but this number in Shenzhen is 10–14 (including 5 ultrasound examinations). The 50–80% prenatal care utilization rate in Shenzhen is approximately equivalent to the 80–100% utilization rate under the WHO guidelines. That is why the stillbirth rate is lowest in the group with 50–80% prenatal care utilization rate in Shenzhen. Due to the influx of a large number of floating population in Shenzhen, it is often difficult for the floating population to follow the official recommendations of prenatal care. They often do not follow the recommended time, or will go to different hospitals for prenatal care. Therefore, too many prenatal care or irregular prenatal care may be the reason for the higher stillbirth rate among the population with more than 80% of prenatal care utilization rate in our analysis. Our results show that the number of prenatal care recommended by the WHO is more reasonable. Further research about a fewer number of prenatal care visits is needed to optimize the allocation of medical resources [46].

## Conclusion

In summary, we determined an overall stillbirth rate of 4.5 stillbirths per 1000 births in a typical internal migrant population in Baoan, Shenzhen, China, during 2010–2019. Migrant women had a lower socioeconomic status, including inadequate prenatal care utilization and a higher risk of stillbirth, than the local population. Migrants from provinces with lower economic levels, usually in the western region, had a lower socioeconomic status and a higher associated risk of stillbirth. Targeting strategies, including sufficient prenatal care service provisions and effective health insurance coverage for migrant women especially from the western China, need to be promoted by health policy makers.

## Supplementary Information

**Additional file 1: Figure S1.** Flow diagram of study selection. **Figure S2.** Maternal sociodemographic characteristics by migration status in Baoan, Shenzhen, 2010–2019. (A) Maternal age, (B) maternal education, (C) GDP per capita group of maternal birthplaces, (D) region of maternal birthplace, (E) trimester of first prenatal care visit and (F) prenatal care utilization group. **Table S1.** Secular trends in stillbirth rate (‰) in Baoan, Shenzhen, 2010–2019. **Table S2.** Distribution percentage (%) of maternal socioeconomic characteristics by migration status in Baoan, Shenzhen, 2010–2019. **Table S3.** Maternal socioeconomic characteristics by maternal birthplace in Baoan, Shenzhen, 2010–2019. **Table S4.** Maternal socioeconomic characteristics by region of maternal birthplace in Baoan, Shenzhen, 2010–2019. **Table S5.** The Spearman’s coefficients between provincial GDP per capita, average maternal age, education level, and prenatal care utilization level for each maternal birthplace in Baoan Shenzhen, 2010–2019.

## Data Availability

The datasets used and/or analyzed during the current study are available from the corresponding author upon request.

## Abbreviations

GDP: Gross domestic product
AOR: Adjusted odds ratio
CI: Confidence interval
