# Supplementary material for: Stillbirth trends by maternal sociodemographic characteristics among a large internal migrant population in Shenzhen, China, over a 10-year period: a retrospective study
Source: BMC Public Health. 2022 Feb 16;22:325. doi: 10.1186/s12889-022-12734-8 (PMC8848954; doi:10.1186/s12889-022-12734-8)
Supplement: Supplementary file 1 — Additional file 1: Figure S1. Flow diagram of study selection. Figure S2. Maternal sociodemographic characteristics by migration status in Baoan, Shenzhen, 2010–2019. (A) Maternal age, (B) maternal education, (C) GDP per capita group of maternal birthplaces, (D) region of maternal birthplace, (E) trimester of first prenatal care visit and (F) prenatal care utilization group. Table S1. Secular trends in stillbirth rate (‰) in Baoan, Shenzhen, 2010–2019. Table S2. Distribution percentage (%) of maternal socioeconomic characteristics by migration status in Baoan, Shenzhen, 2010–2019. Table S3. Maternal socioeconomic characteristics by maternal birthplace in Baoan, Shenzhen, 2010–2019. Table S4. Maternal socioeconomic characteristics by region of maternal birthplace in Baoan, Shenzhen, 2010–2019. Table S5. The Spearman’s coefficients between provincial GDP per capita, average maternal age, education level, and prenatal care utilization level for each maternal birthplace in Baoan Shenzhen, 2010–2019. [file 12889_2022_12734_MOESM1_ESM.docx]

## Stillbirth trends by maternal sociodemographic characteristics among a large internal migrant population in Shenzhen, China, over a 10-year period: a retrospective study


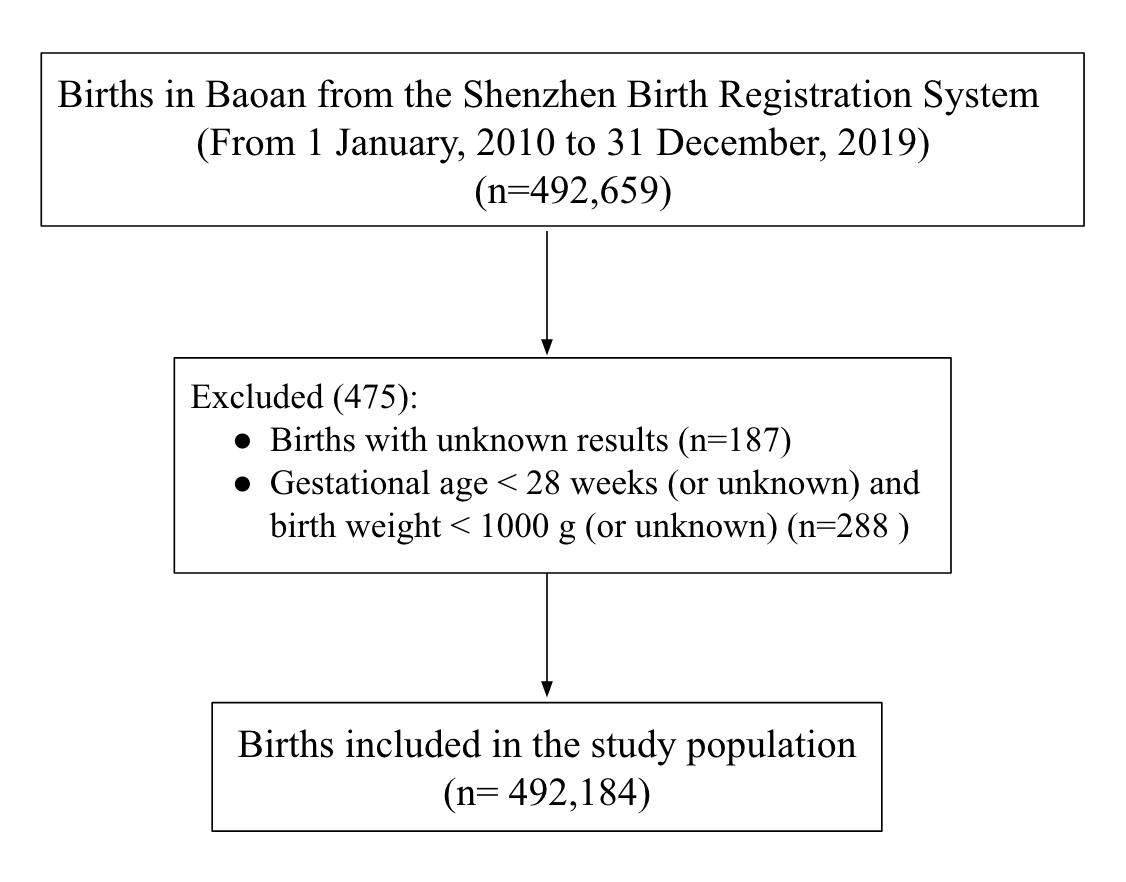


**Figure S1**. Flow diagram of study selection


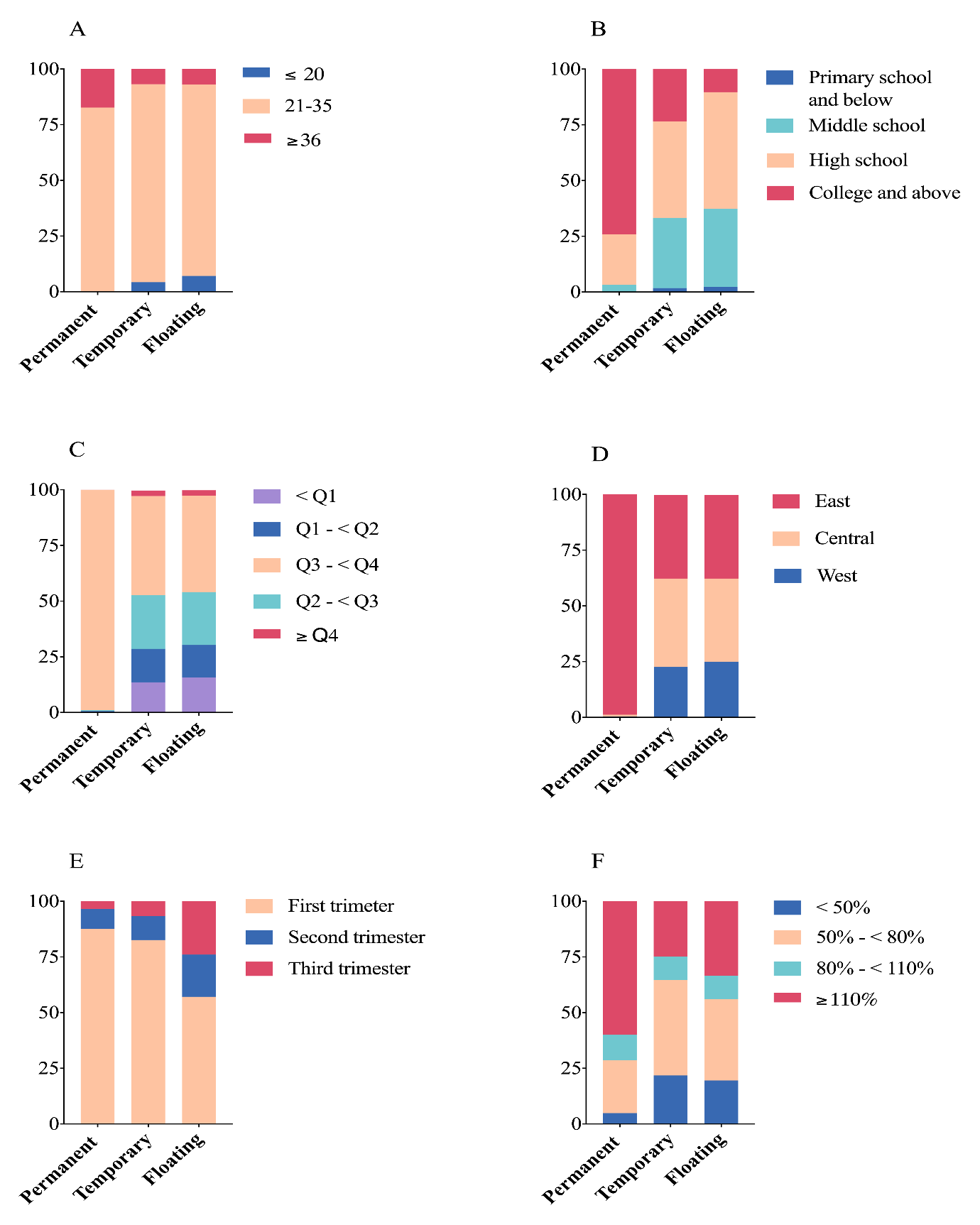


**Figure S2**. Maternal sociodemographic characteristics by migration status in Baoan, Shenzhen, 2010–2019. (A) Maternal age, (B) maternal education, (C) GDP per capita group of maternal birthplaces, (D) region of maternal birthplace, (E) trimester of first prenatal care visit and (F) prenatal care utilization group.

**Table S1.** Secular trends in stillbirth rate (‰) in Baoan, Shenzhen, 2010 - 2019

|  | **Year** | | | | | | | | | |
| --- | --- | --- | --- | --- | --- | --- | --- | --- | --- | --- |
|  | 2010 | 2011 | 2012 | 2013 | 2014 | 2015 | 2016 | 2017 | 2018 | 2019 |
| **Overall** | 4.52 | 4.84 | 4.34 | 4.03 | 4.69 | 4.66 | 4.15 | 3.91 | 5.12 | 5.10 |
| **Maternal age (year)** |  |  |  |  |  |  |  |  |  |  |
| ≤ 20 | 8.52 | 5.58 | 5.96 | 6.85 | 5.49 | 8.70 | 5.72 | 7.37 | 10.07 | 11.90 |
| 21-35 | 4.20 | 4.47 | 4.17 | 3.57 | 4.51 | 4.30 | 3.89 | 3.66 | 4.71 | 4.07 |
| ≥ 36 | 4.79 | 6.48 | 5.90 | 6.95 | 5.59 | 6.46 | 6.21 | 5.88 | 5.60 | 6.50 |
| **Maternal education** |  |  |  |  |  |  |  |  |  |  |
| Primary school and below | 5.49 | 5.88 | 6.15 | 9.06 | 5.10 | 8.25 | 5.93 | 9.24 | 13.75 | 9.96 |
| Middle school | 4.52 | 4.65 | 4.01 | 5.31 | 4.86 | 4.69 | 5.02 | 5.71 | 8.93 | 8.67 |
| High school | 5.22 | 5.40 | 5.05 | 3.48 | 5.16 | 5.32 | 4.50 | 3.90 | 5.08 | 6.98 |
| College and above | 2.62 | 3.11 | 2.98 | 2.68 | 3.38 | 3.15 | 2.92 | 2.69 | 3.16 | 2.50 |
| **Migration status** |  |  |  |  |  |  |  |  |  |  |
| Permanent | 2.62 | 2.12 | 2.54 | 3.61 | 3.70 | 2.36 | 3.21 | 2.70 | 2.68 | 2.48 |
| Temporary | 4.18 | 4.11 | 3.63 | 3.50 | 4.35 | 4.32 | 4.25 | 4.21 | 5.18 | 5.74 |
| Floating | 4.86 | 5.84 | 5.52 | 5.65 | 6.18 | 6.68 | 5.78 | 6.56 | 8.53 | 7.99 |
| **GDP group of**  **maternal birthplace^*^** |  |  |  |  |  |  |  |  |  |  |
| < Q1 | 5.74 | 6.40 | 5.63 | 4.88 | 6.23 | 6.49 | 5.04 | 5.57 | 10.29 | 7.55 |
| Q1 - < Q2 | 5.26 | 4.98 | 5.02 | 4.98 | 5.25 | 5.17 | 4.15 | 4.63 | 7.11 | 5.77 |
| Q2 - < Q3 | 4.55 | 4.65 | 4.43 | 3.87 | 4.18 | 4.03 | 4.34 | 3.81 | 5.90 | 5.40 |
| Q3 - < Q4 | 3.85 | 4.51 | 4.37 | 4.16 | 4.33 | 4.23 | 4.01 | 3.55 | 3.98 | 4.06 |
| ≥ Q4 | 3.54 | 2.47 | 3.06 | 3.12 | 3.51 | 3.98 | 2.01 | 2.05 | 3.59 | 2.63 |
| **Time of first visit** |  |  |  |  |  |  |  |  |  |  |
| First trimester | 3.86 | 4.17 | 3.47 | 3.24 | 3.55 | 3.76 | 3.37 | 3.51 | 4.58 | 3.55 |
| Second trimester | 4.22 | 4.46 | 5.63 | 6.13 | 7.88 | 8.46 | 6.23 | 6.17 | 8.62 | 6.17 |
| Third trimester | 6.01 | 6.52 | 6.84 | 10.23 | 14.10 | 11.20 | 13.91 | 12.35 | 13.28 | 11.89 |
| **Prenatal care utilisation**^†^ |  |  |  |  |  |  |  |  |  |  |
| < 50% | 5.01 | 6.63 | 7.39 | 11.17 | 13.51 | 14.83 | 11.66 | 10.98 | 14.06 | 12.39 |
| 50% - < 80% | 3.65 | 2.12 | 1.48 | 1.26 | 1.78 | 1.14 | 1.60 | 1.13 | 2.21 | 3.43 |
| 80% - < 110% | 4.39 | 3.86 | 4.60 | 2.92 | 4.10 | 4.27 | 3.47 | 4.13 | 5.89 | 5.61 |
| ≥ 110% | 4.68 | 7.17 | 6.56 | 6.17 | 5.36 | 6.10 | 5.35 | 4.75 | 7.52 | 4.80 |

*. GDP group of maternal birthplace is generated by classifying maternal birthplace by the average GDP per capita during 2010 and 2019.

†. Prenatal care utilisation rate is defined as the ratio between the actual number of visits and the recommended number.

**Table S2.** Distribution percentage (%) of maternal socioeconomic characteristics by migration status in Baoan, Shenzhen, 2010 - 2019

|  | **Permanent Population**  **N(%)** | **Temporary Population**  **N(%)** | **Floating Population**  **N(%)** |
| --- | --- | --- | --- |
| **Total** | 64439 | 293842 | 133903 |
| **Maternal age (year)** |  |  |  |
| ≤ 15 | 13(0.02) | 199(0.1) | 150(0.1) |
| 16-20 | 156(0.2) | 12201(4.2) | 9396(7.0) |
| 21-35 | 53153(82.5) | 261244(88.9) | 115141(86) |
| ≥ 36 | 11117(17.3) | 20198(6.9) | 9216(6.9) |
| **Maternal education** |  |  |  |
| Primary school and below | 156(0.2) | 5314(1.8) | 3223(2.4) |
| Middle school | 1913(3.03) | 92487(31.5) | 46674(34.9) |
| High school | 14618(22.7) | 126858(43.2) | 70167(52.4) |
| College and above | 47752(74.1) | 69183(23.5) | 13839(10.3) |
| **GDP group of**  **maternal birthplace**^*^ |  |  |  |
| < Q1 | 171(0.3) | 39922(13.6) | 20990(15.7) |
| Q1 - < Q2 | 165(0.3) | 44066(15.0) | 19528(14.6) |
| Q2 - < Q3 | 312(0.5) | 70666(24.0) | 31818(23.8) |
| Q3 - < Q4 | 63768(99.0) | 131062(44.6) | 58018(43.3) |
| ≥ Q4 | 20(0.03) | 7412(2.5) | 3265(2.4) |
| Missing | 3(0.0) | 714(0.2) | 284(0.2) |
| **Region of maternal birthplace** |  |  |  |
| West | 262(0.4) | 66289(22.6) | 33426(25.0) |
| Central | 497(0.8) | 116663(39.7) | 49995(37.3) |
| East | 63677(98.8) | 110176(37.5) | 50198(37.5) |
| Missing | 3(0.0) | 714(0.2) | 284(0.2) |
| **Time of first visit** |  |  |  |
| First trimester | 56453(87.6) | 242638(82.6) | 76421(57.1) |
| Second trimester | 5783(9.0) | 31619(10.8) | 25281(18.9) |
| Third trimester | 2203(3.4) | 19585(6.7) | 32201(24.0) |
| **Prenatal care utilisation**^†^ |  |  |  |
| < 50% | 3171(4.9) | 46872(16) | 56241(42.0) |
| 50% - < 80% | 15244(23.7) | 133071(45.3) | 50559(37.8) |
| 80% -< 110% | 7351(11.4) | 32731(11.1) | 10580(7.9) |
| ≥ 110% | 38673(60) | 81168(27.6) | 16523(12.3) |

*. GDP group of maternal birthplace is generated by classifying maternal birthplace by the average GDP per capita during 2010 and 2019.

†. Prenatal care utilisation rate is defined as the ratio between the actual number of visits and the recommended number.

**Table S3.** Maternal socioeconomic characteristics by maternal birthplace in Baoan, Shenzhen, 2010 - 2019

| **Province** | **Temporary and floating population** | **Average GDP**  **per Capita ($)**^*^ | **Stillbirth**  **rate (‰)**^†^ | **Average age**  **(year)** | **College and**  **above rate (%)**^‡^ | **Intermediate and above rate (%)**^§^ |
| --- | --- | --- | --- | --- | --- | --- |
| Beijing | 132 | 16796.3 | NA | 32.3 | 71.2 | 93.3 |
| Shanghai | 65 | 16425.4 | NA | 33.3 | 66.7 | 95.6 |
| Tianjin | 116 | 15564.4 | NA | 31.5 | 70.9 | 92.4 |
| Jiangsu | 1514 | 13367.8 | 1.30 | 29.6 | 36.0 | 85.6 |
| Zhejiang | 1487 | 11923.7 | 2.32 | 30.5 | 30.0 | 86.3 |
| Fujian | 7335 | 10504.4 | 2.66 | 29.2 | 26.7 | 75.1 |
| Guangdong | 142950 | 10355.3 | 3.85 | 28.8 | 33.9 | 82.0 |
| Inner Mongolia | 478 | 10047.7 | 4.12 | 29.7 | 39.3 | 65.4 |
| Shandong | 2681 | 9389.8 | 3.16 | 29.9 | 34.5 | 81.7 |
| Liaoning | 715 | 8683.8 | 4.09 | 30.2 | 33.7 | 75.6 |
| Chongqing | 6964 | 7791.2 | 3.72 | 27.5 | 32.3 | 74.3 |
| Hubei | 35143 | 7683.1 | 3.78 | 28.7 | 38.6 | 86.1 |
| Shaanxi | 5927 | 7296.3 | 4.00 | 28.6 | 26.3 | 84.2 |
| Jilin | 878 | 7246.5 | 4.56 | 30.2 | 30.7 | 69.7 |
| Ningxia | 128 | 6574.6 | NA | 28.9 | 28.1 | 51.5 |
| Hunan | 70755 | 6363.2 | 4.39 | 28.2 | 34.3 | 69.2 |
| Hebei | 1098 | 6168.7 | 2.70 | 29.3 | 31.5 | 83.4 |
| Hainan | 2127 | 6166.3 | 5.60 | 28.8 | 23.3 | 67.1 |
| Xinjiang | 264 | 6092.6 | NA | 28.2 | 25.2 | 63.2 |
| Henan | 21724 | 5995.5 | 4.69 | 28.1 | 24.2 | 72.3 |
| Qinghai | 340 | 5980.9 | 8.77 | 26.2 | 15.2 | 45.6 |
| Sichuan | 26782 | 5686.2 | 5.74 | 27.3 | 23.9 | 64.4 |
| Anhui | 7504 | 5624.3 | 4.53 | 28.4 | 23.6 | 73.3 |
| Shanxi | 629 | 5608.5 | 4.71 | 29.0 | 34.8 | 74.1 |
| Heilongjiang | 1430 | 5762.6 | NA | 29.9 | 30.6 | 72.6 |
| Jiangxi | 28676 | 5583.6 | 4.62 | 28.1 | 25.9 | 71.9 |
| Guangxi | 38831 | 5120.3 | 6.00 | 27.9 | 22.9 | 73.6 |
| Tibet | 9 | 4817.3 | NA | 28.3 | 10.4 | 45.7 |
| Yunnan | 3603 | 4428.9 | 6.37 | 26.1 | 18.8 | 51.0 |
| Guizhou | 14316 | 4402.6 | 6.79 | 26.2 | 20.1 | 52.6 |
| Gansu | 2131 | 3926.3 | 8.88 | 27.6 | 21.9 | 47.8 |

*. Average GDP per capita for each maternal birthplace was calculated by the sum of annual GDP per capita divided by 10 years.

†. NA:Stillbirth rates of material birthplace with less than 300 migrants or 1 stillbirth were excluded.

‡. Percentage of college and above maternal education.

§. Percentage of intermediate and above prenatal care utilisation.

**Table S4.** Maternal socioeconomic characteristics by region of maternal birthplace in Baoan, Shenzhen, 2010-2019

|  | **Region of maternal birthplace** | | |
| --- | --- | --- | --- |
|  | **West** | **Central** | **East** |
| **Stillbirth rate (‰)** | 5.7 | 4.3 | 4.1 |
| **Maternal age (year)** |  |  |  |
| ≤ 20 | 8.1 | 3.9 | 3.4 |
| 21-35 | 84.8 | 89.1 | 87.0 |
| ≥ 36 | 7.1 | 7.0 | 9.6 |
| **Maternal education** |  |  |  |
| Primary school and below | 2.8 | 1.2 | 1.7 |
| Middle school | 39.7 | 27.6 | 24.6 |
| High school | 43.9 | 46.0 | 40.2 |
| College and above | 13.6 | 25.2 | 33.5 |
| **Migration status** |  |  |  |
| Permanent population | 0.3 | 0.3 | 28.4 |
| Temporary population | 66.3 | 69.8 | 49.2 |
| Floating population | 33.4 | 29.9 | 22.4 |
| **GDP group of**  **maternal birthplace^*^** |  |  |  |
| < Q1 | 58.9 | 1.2 | 0.0 |
| Q1 - < Q2 | 27.5 | 21.7 | 0.0 |
| Q2 - < Q3 | 6.1 | 55.9 | 1.4 |
| Q3 - < Q4 | 7.5 | 21.2 | 93.8 |
| ≥ Q4 | 0.0 | 0.0 | 4.8 |
| **Time of first visit** |  |  |  |
| First trimester | 74.2 | 75.6 | 77.7 |
| Second trimester | 12.7 | 13.2 | 12.5 |
| Third trimester | 13.1 | 11.2 | 9.8 |
| **Prenatal care utilisation^†^** |  |  |  |
| < 50% | 25.7 | 21.8 | 19.5 |
| 50% - < 80% | 44.9 | 42.8 | 36.6 |
| 80% - < 110% | 9.8 | 10.5 | 10.4 |
| ≥ 110% | 19.6 | 24.9 | 33.5 |

*. GDP group of maternal birthplace is generated by classifying maternal birthplace by the average GDP per capita during 2010 and 2019.

†. Prenatal care utilisation rate is defined as the ratio between the actual number of visits and the recommended number.

**Table S5.** The Spearman's coefficients between provincial GDP per capita, average maternal age, education level, and prenatal care utilization level for each maternal birthplace in Baoan Shenzhen, 2010–2019.

|  | **Spearman's coefficient** | ***P* value** |
| --- | --- | --- |
| Average age | 0.754 | <0.001 |
| Education level^*^ | 0.792 | <0.001 |
| Prenatal care utilization level^†^ | 0.751 | <0.001 |

*. Provincial education level was a percentage calculated by births with college and above maternal education divided by total birth in each province.

†. Prenatal care utilization level was a percentage calculated by births with intermediate and above maternal prenatal care utilization rate (≥50%) divided by total births in each province.

‡. 24 provinces were included to calculation of stillbirth rate with the criteria: no less than 300 births and 1 stillbirth.
